# Iodine Concentration in Drinking Water in the Same or Different Seasons of the Year in Brazilian Macroregions

**DOI:** 10.1155/2022/7227511

**Published:** 2022-08-04

**Authors:** Carina Aparecida Pinto, Dayane de Castro Morais, Sylvia do Carmo Castro Franceschini, Sarah Aparecida Vieira Ribeiro, Edimar Aparecida Filomeno Fontes, Nathalia Marcolini Pelucio Pizato, Franciane Rocha de Faria, Renata Junqueira Pereira, Danielle Goés da Silva, Carolina Abreu de Carvalho, Mariana de Souza Macedo, Naiara Sperandio, Anderson Marliere Navarro, Sandra Patrícia Crispim, Silvia Eloiza Priore

**Affiliations:** ^1^ Department of Nutrition and Health Universidade Federal de Viçosa (UFV) Viçosa 36570.900, Brazil, ufv.br; ^2^ Department of Food Technology Universidade Federal de Viçosa (UFV) Viçosa 36570.900, Brazil, ufv.br; ^3^ Department of Nutrition Universidade de Brasília (UnB) Brasília 70910.900, Brazil, unb.br; ^4^ Departament of Medicine Universidade Federal de Rondonópolis (UFR) Rondonópolis 78735.910, Brazil; ^5^ Department of Health Sciences Universidade Federal do Tocantis (UFT) Palmas 77001.090, Brazil; ^6^ Department of Nutrition Universidade Federal do Sergipe (UFS) Aracaju 49100.000, Brazil; ^7^ Department of Medicine Universidade Federal do Maranhão (UFMA) São Luís 65200.000, Brazil, ufma.br; ^8^ Department of Nutrition Federal University of the Jequitinhonha and Mucuri Valleys Diamantina, Brazil, ufvjm.edu.br; ^9^ Institute of Food and Nutrition Universidade Federal do Rio de Janeiro (UFRJ) Macaé 27930.560, Brazil, ufrj.br; ^10^ Department of Health Sciences Universidade de São Paulo (USP) Ribeirão Preto 14040.900, Brazil, usp.br; ^11^ Department of Nutrition Universidade Federal do Paraná (UFPR) Curitiba 80210.170, Brazil, ufpr.br

## Abstract

**Objective:**

The aim of this study was to compare the concentration of iodine in drinking water in the same or different seasons of the year in Brazilian macroregions.

**Method:**

Water samples were collected from the Basic Health Units of eight municipalities that make up the different Brazilian macroregions and the Federal District. Sample collection took place in the summer, autumn, winter, and spring seasons. The spectrophotometric method with “leuco crystal violet” was used to determine the concentration of iodine in the water. Descriptive statistics on the data were performed. To verify if there was a difference in the concentration of iodine in the water between the climatic seasons of the year in the same place and between the same seasons in different locations, the Mann–Whitney or Kruskal–Wallis test was used and a *p* < 0.05 value was considered significant.

**Results:**

Among the climatic seasons throughout the year in the same location, there was a difference in the concentration of iodine in the water in the municipality of Pinhais, state of Paraná/South macroregion, between autumn and summer (*p* = 0.041) and winter and summer seasons (*p* = 0.003). There was a difference in the concentration of iodine in the water in the summer season between the Midwest and South macroregions; Northeast and Midwest, Southeast and South; North and Midwest, Southeast and South (*p* < 0.05). In the autumn season, there was a difference in the concentration of iodine in the water between the Midwest and South macroregions; Northeast and Midwest, Southeast and South; North and Midwest, Northeast and South (*p* < 0.05). In the winter season, there was a difference in the concentration of iodine in the water between the Southeast and Midwest and Southeast and South macroregions (*p* < 0.05). In the spring season, there was a difference in the concentration of iodine in the water between the Southeast and Midwest and Southeast and South macroregions (*p* < 0.05).

**Conclusion:**

There were differences in the iodine concentrations in drinking water in different locations in Brazil, when analyzed in the same seasons, and in the municipality of Pinhais between the autumn and summer and winter and summer seasons. Thus, it is suggested to monitor the iodine concentrations in water, considering the differences in climate, characteristics of each region, and soils throughout the Brazilian territory, since the deficiency or excess of iodine can bring risks to the health of the population.

## 1. Introduction

Iodine is an indispensable nutrient for the synthesis of the thyroid hormones, triiodothyronine and thyroxine, necessary for normal growth, development, and metabolism during pregnancy, childhood, and throughout life [1–3].

Deficiency or excess of iodine in the body is responsible for health problems such as hypothyroidism, hypothyroxinemia, goiter, cretinism, decreased fertility, increased prenatal and infant mortality, hyperthyroidism, thyroiditis, and autoimmune thyroid diseases [1, 4, 5].

Iodine is present in water in concentrations that vary according to the mineral content in the soils of the region [6]. In turn, the iodine content in the soil can vary within and between regions due to several factors, including differences that occurred during geological formation, soil types, climatic conditions, distance from the sea, flooding, soil erosion, and application of fertilizers [6, 7].

Regarding climatic conditions, these are decisive for the definition of the seasons in summer, autumn, winter and spring. Each climatic season presents peculiar and defined characteristics. However, the seasons of the year do not occur homogeneously in the five macroregions (Central West, Northeast, North, Southeast, and South) that are part of the entire Brazilian territory [8].

The states of Paraná, Santa Catarina, and Rio Grande do Sul (South macroregion); São Paulo, Minas Gerais, and Rio de Janeiro (Southeast macroregion); and Mato Grosso do Sul (one of the states that are part of the Center‐West macroregion) have the most well‐defined climatic seasons during the year. In the other Brazilian macroregions (mainly Northeast and North), there are basically two well‐defined seasons: one hot and humid, the other hot and dry [8], depending on the volume and frequency of precipitation in these locations.

Thus, considering that each location in each Brazilian macroregion has seasons with specific characteristics, this study proposes to compare the concentration of iodine in drinking water in the same or different climatic seasons of the year in Brazilian macroregions.

## 2. Materials and Methods

This study is part of the project called “Nutritional Status of Iodine, Sodium, and Potassium in the Brazilian Maternal‐Infant Group: a Multicenter Study,” which was approved by the Ethics Committee in Research with Human Beings of the Federal University of Viçosa with opinion number 2,496,986.

Water samples were collected at the Basic Health Units of eight municipalities and the Federal District, covering the five Brazilian macroregions (Midwest, Northeast, North, Southeast and South). Sample collection took place during the summer seasons (from 12/21/2018 to 03/19/2019), autumn (from 03/20/2019 to 06/19/2019), winter (from 06/20/2019 to 21/09/2019) and spring (from 22/09/2019 to 20/12/2019).

The number of water samples collected in each location and in each season of the year is shown in Table 1.

**Table 1:** Location, macroregion and number of water samples collected by the climate station from Brazil.

Locality/Brazilian macroregion	Number of water samples per season			
	Summer	Autumn	Winter	Spring
Brasília‐DF/Midwest^1^	10	10	10	—
Rondonópolis/Midwest	12	12	12	12
Aracaju/Northeast^1^	12	12	—	12
São Luís/Northeast^1^	12	12	—	—
Palmas/North^1^	10	10	—	—
Macaé/Southeast^1^	9	—	—	—
Ribeirão Preto/Southeast^1^	—	—	9	9
Viçosa/Southeast	14	14	14	14
Pinhais/South	11	11	11	11

^1^No water samples were collected in all seasons at these locations. DF = Federal District.

The water samples were collected by the team from each location and stored in freezers (−18°C) in each respective research center in each macroregion and later sent to the accredited laboratory for the analysis. The samples were transported frozen in thermal boxes and, in the research laboratory of chemistry and food analysis of the Department of Food Technology at the Federal University of Viçosa; they were kept at a temperature of 4°C until the moment of analysis.

The spectrophotometric method “leuco crystal violet” was used to determine the concentration of iodine in drinking water. This method was based on Standard Methods for the Examination of Water and Wastewater, 4500‐IB [9].

The analysis was performed in triplicate of each sample, and the average concentration of iodine was obtained and expressed in g of iodine L^−1^.

Data analysis was performed using the Statistical Program for Social Science (SPSS) version 21.0. Descriptive statistics were performed, and the median, minimum, and maximum values were used to describe the concentration of iodine in the water.

To verify if there was a difference in the concentration of iodine in drinking water between the climatic seasons of the same location and between the same seasons in different locations, the Mann–Whitney or Kruskal–Wallis test was applied, according to the normality of the data, and a considered significant *p* value 0.05 was found.

## 3. Results

No difference was observed in the concentration of iodine in drinking water between the climatic seasons in the municipalities of Aracaju (Sergipe State/Northeast macroregion) (*p* = 0.197), Ribeiro Preto (São Paulo State/Southeast macroregion) (*p* = 0.208), São Luís (State of Maranhão/Northeast macroregion) (*p* = 0.255), Palmas (State of Tocantins/North macroregion) (*p* = 0.327), Viçosa (State of Minas Gerais/Southeast macroregion) (*p* = 0.340), Brasília (Fedwest macroregion) (*p* = 0.548), and Rondonópolis (Mato Grosso State/Midwest macroregion) (*p* = 1.000). It is noteworthy that this analysis was not carried out for the municipality of Macaé (State of Rio de Janeiro/Southeast macroregion) due to the absence of water samples from all climatic seasons. There was a difference in the concentration of iodine in drinking water between the autumn and summer seasons (*p* = 0.041) and winter and summer (*p* = 0.003) in the municipality of Pinhais (State of Paraná/South macroregion) (Table 2).

**Table 2:** Comparison of iodine concentration in drinking water between climatic seasons in the same location.

Locality/Brazilian macroregion	Median iodine concentration (*μ*g/L)			
	Water seasons			
	Summer	Autumn	Winter	Spring
Brasília‐DF/Midwest	4.43^a^	3.81^a^	4.92^a^	—
Rondonópolis/Midwest	2.33^a^	0.00^a^	3.04^a^	3.04^a^
Aracaju/Northeast	3.55^a^	2.76^a^	—	3.55^a^
São Luís/Northeast	0.00^a^	0.00^a^	—	0.19^a^
Palmas/North	0.41^b^	0.00^b^	—	—
Ribeirão Preto/Southeast	—	—	0.96^b^	0.09^b^
Viçosa/Southeast	3.33^a^	2.49^a^	1.64^a^	3.75^a^
Pinhais/South	13.04^a^	5.45^c^	4.60^c^	7.98^a^

^a^Median followed by equal letters do not differ (*p* > 0.05) by the Kruskal–Wallis test. ^b^Median followed by equal letters do not differ (*p* > 0.05) by the Mann–Whitney test. a,cMedian followed by different letters differ (*p* < 0.05) by the Kruskal–Wallis test. DF = Federal District.

In the summer season, the median concentration of iodine in water ranged from 0.00 *μ*g/L to 13.04 *μ*g/L among the different Brazilian locations (Table 3). We observed difference in water iodine concentration between Rondonópolis and Pinhais (*p* = 0.001); São Luís and Pinhais (*p* < 0.0001); São Luís and Brasília (*p* = 0.015); São Luís and Viçosa (*p* = 0.030); Palmas and Pinhais (*p* < 0.0001); Palmas and Brasília (*p* = 0.016); and Palmas and Viçosa (*p* = 0.031).

**Table 3:** Median, minimum and maximum concentration of iodine in water samples collected in Basic Health Units (UBS) in the different climatic seasons of the places belonging to the Brazilian macroregions.

Climate season	Median iodine concentration (min‐max) (*μ*g·L^−1^)								
	Locations/Brazilian macroregions								
	Midwest		Notheast		North	Southeast			South
	Brasília	Rondonópolis	Aracaju	São Luís	Palmas	Macaé	Ribeirão Preto	Viçosa	Pinhais
Summer	4.43 (2.58–18.58)	2.33 (0.00–10.59)	3.55 (1.18–5.91)	0.00 (0.00–4.88)	0.41 (0.00–2.68)	3.85 (0.55–24.18)	—	3.33 (0.38–28.24)	13.04 (5.45–42.59)
Autumn	3.81 (2.58–6.28)	0.00 (0.00–113.86)	2.76 (1.18–5.91)	0.00 (0.00–1.93)	0.00 (0.00–2.68)	—	—	2.49 (0.38–12.20)	5.45 (2.91–11.35)
Winter	4.92 (2.39–6.37)	3.04 (1.30–109.14)	—	—	—	—	0.96 (0.09–2.70)	1.64 (0.38–10.51)	4.60 (2.91–20.64)
Spring	—	3.04 (1.30–13.48)	3.55 (1.18–8.28)	—	—	—	0.09 (0.00–4.45)	3.75 (0.38–17.26)	7.98 (2.91–20.64)

The median iodine concentration in water ranged from 0.00 *μ*g/L to 5.45 *μ*g/L in the fall season among the different Brazilian locations (Table 3). It was different between water from Rondonópolis and Pinhais (*p* = 0.007); Palmas and Pinhais (*p* < 0.0001); Palmas and Brasília (*p* = 0.014); Palmas and Aracaju (*p* = 0.044); São Luís and Pinhais (*p* < 0.0001); São Luís and Brasília (*p* = 0.002); São Luís and Aracaju (*p* = 0.007); and São Luís and Viçosa (*p* = 0.029).

In the winter season, the median concentration of iodine in water ranged from 0.96 *μ*g/L to 4.92 *μ*g/L and there were differences between Ribeirão Preto and Brasília (*p* = 0.011); Ribeirão Preto and Rondonópolis (*p* = 0.006); and Ribeirão Preto and Pinhais (*p* = 0.002) (Table 3).

The median iodine concentration in water ranged from 0.09 *μ*g/L to 7.98 *μ*g/L among the different Brazilian locations in the spring season (Table 3). It was different between water from Ribeirão Preto and Rondonópolis (*p* = 0.041); and Ribeirão Preto and Pinhais (*p* < 0.001).

## 4. Discussion

Differences were observed in the concentration of iodine in the drinking water of the different locations, due to the fact that Brazil is a country of continental dimensions.

The concentration of iodine in drinking water showed different results between the climatic seasons in the municipality of Pinhais, located in the interior of the state of Paraná (South). In this location, a higher median concentration of iodine in the water was observed in summer than in autumn and winter.

In the municipality of Pinhais, the month of January, which corresponds to the summer season, is the wettest month with approximately 233 millimeters of precipitation, while the months of April and August, which correspond to the autumn and winter seasons, respectively, have both 84 millimeters of precipitation [10, 11]. According to these characteristics, a lower median concentration of iodine in the water in the summer season was expected because a greater volume of precipitation causes an increase in the water level in the reservoirs, resulting in the dilution and lower concentration of the mineral in the water [12].

The concentration of iodine in drinking water also showed different results in the same climatic seasons between the different locations of the Brazilian macroregions (Midwest, Northeast, North, Southeast, and South).

In the summer season, it was found that the drinking water of São Luís (Northeast macroregion) had a lower median concentration of iodine in relation to Brasília (Central West macroregion), Viçosa (Southeast macroregion), and Pinhais (South macroregion). In São Luís, the month of March, referring to the summer season, is the month with the highest precipitation with an average of 440 millimeters, a value well above the average of Brasília (217 millimeters), Viçosa (136 millimeters), and Pinhais forests (137 mm) for the same month. This greater volume of precipitation in São Luís contributes to the increase in the water level in the reservoirs, leading to dilution and consequently a lower concentration of the minerals in the water of this location [10, 12].

In the summer season, it was also observed that the drinking water of Palmas (Central West macroregion) had a lower median concentration of iodine in relation to that of Brasília (Central West macroregion), Viçosa (Southeast macroregion), and Pinhais (South macroregion). The municipality of Palmas during the summer season has an average of 332 millimeters of rain, while Brasília, Viçosa, and Pinhais have an average of 216 millimeters, 158 millimeters, and 189 millimeters of rain, respectively. In addition, in Palmas, the average temperature (25.6°C) during the summer season is higher than in Brasília (22.1°C), Viçosa (22.6°C), and Pinhais (20.4°C) [10]. A greater volume of precipitation and high temperatures are factors that contribute to the lower concentration of iodine in the water because there is an increase in the level of water in the reservoirs, allowing the dilution of the mineral [12].

In the autumn season, it was found that the drinking water of São Luís (Northeast Macroregion) had a lower median concentration of iodine in relation to that of Brasília (Central West macroregion), Aracaju (Northeast macroregion), Viçosa (Southeast macroregion), and Pinhais (South Macroregion). The higher volume of precipitation in São Luís during the autumn season, with an average of 291 millimeters, explains the lower concentration of iodine in the water at this location compared to Brasília (average of 44 millimeters of rain), Aracaju (average of 168 millimeters of rain), Viçosa (average of 40 millimeters of rain), and Pinhais (average of 95 millimeters of rain) [10].

In the autumn season, it was also observed that the drinking water of Palmas (Central West macroregion) had a lower median concentration of iodine in relation to that of Brasília (Central West macroregion), Aracaju (Northeast macroregion), and Pinhais (South Macroregion). At Palmas we had a higher average temperature (26.5°C) during the autumn season than at other locations (Brasília, Aracaju, and Pinhais), and this environmental factor may have contributed to the evaporation of iodine, since it is more volatile at room temperature [6, 10].

In the winter season, it was found that the drinking water of Ribeirão Preto (Southeast macroregion) had a lower median concentration of iodine in relation to that of Brasília (Central West macroregion), Rondonópolis (Central West macroregion), and Pinhais (South macroregion). High temperatures and a greater volume of precipitation contribute to reducing the concentration of iodine in the water [12]. However, in the municipality of Ribeirão Preto during the winter season, there was no high temperature (21.7°C) and a greater volume of precipitation (38 mm) than at other locations, so other factors may be interfering with the iodine concentration in the water [10].

In the spring season, it was observed that the drinking water of Ribeirão Preto (Southeast macroregion) had a lower median concentration of iodine in relation to that of Rondonópolis (Central West macroregion) and Pinhais (South macroregion). However, in the winter season, other factors may be contributing to the lower concentration of iodine in the water of Ribeirão Preto than in other places.

The strengths of this study are the analysis of iodine concentration in drinking water from different locations belonging to the five Brazilian macroregions. In addition to mapping the iodine concentration in Brazilian drinking water, the influence of the climatic season on the concentration of iodine in the water between different places or in the same place was evaluated, highlighting the importance of considering this factor. As a limitation, it is mentioned that it was not possible to collect samples of drinking water in all climatic seasons from some places.

In addition, the absence of studies carried out in the country, as well as the existing differences according to location and climatic factors, make comparison with other studies difficult.

## 5. Conclusions

Differences in iodine concentrations in the water were observed between the different locations of the Brazilian macroregions in different climatic seasons. Therefore, it is suggested to monitor the concentration of iodine in the water considering the climatic seasons of the year, as well as the characteristics of each region and soil types since the deficiency or excess of iodine can pose risks to the health of the population.

## Conflicts of Interest

The authors declare no conflicts of interest.

## Authors’ Contributions

C. A. P and S. E. P designed the study; C. A. P performed the study; C. A. P, D. C. M, and S. E. P analyzed and interpreted the data. C. A. P wrote the article. All authors provided critical conceptual contributions and critically reviewed the manuscript. All authors approved the final manuscript.

## Supporting information


**Supplementary Materials** Two files are included in “Supplementary Files.” The files refer to the statistical analyses used to compare the concentration of iodine in drinking water between the climatic seasons of the same location and in the same climatic seasons between different locations. The file entitled “Results of Article Part 1‐Statistical Analysis” shows the comparison tests used to compare the concentration of iodine in drinking water between the climatic seasons of the same location. The other file entitled “Results of Article Part 2‐Statistical Analysis” shows the comparison tests used to compare the concentration of iodine in drinking water in the same climate stations between different locations in Brazilian macroregions.

## Data Availability

Data are available in Excel spreadsheets.

## References

[1] World health organization/United nations children’s fund/international council for the control of iodine deficiency disorders , Assessment of Iodine Deficiency Disorders and Monitoring Their Elimination: A Guide for Programme Managers, 2007, 3rd edition, World Health Organization, Geneva, Switzerland.

[2] Zimmermann M. B. , Iodine deficiency, Endocrine Reviews. (2009) 30, no. 4, 376–408, 10.1210/er.2009-0011. 19460960

[3] Machamba A. A. L. , Azevedo F. M. , Fracalossi K. O. , and Franceschini S. d. C. C. , Effect of iodine supplementation in pregnancy on neurocognitive development on offspring in iodine deficiency areas: a systematic review, Archives of Endocrinology and Metabolism. (2021) 65, no. 3, 352–367, 10.20945/2359-3997000000376. 34191411 PMC10065350

[4] Brasil Ministério da Saúde and UNICEF , Cadernos de Atenção Básica: Carência de Micronutrientes, 2007, Ministério da Saúde, Brasília, Brazil.

[5] Opazo M. C. , Coronado-Arrázola I. , Vallejos O. P. , Moreno-Reyes R. , Fardella C. , Mosso L. , Kalergis A. M. , Bueno S. M. , and Riedel C. A. , The impact of the micronutrient iodine in health and diseases, Critical Reviews in Food Science and Nutrition. (2020) 62, no. 6, 1466–1479, 10.1080/10408398.2020.1843398. 33226264

[6] Lima L. F. and Navarro A. M. , Funções plenamente reconhecidas de nutrientes-iodo/ISLI Brasil. Série de publicações ISLI Brasil: funções plenamente reconhecidas de nutrientes, Força-Tarefa de Alimentos Fortificados e Suplementos. (2018) 22, 1–33.

[7] Zimmermann M. B. , Symposium on “geographical and geological influences on nutrition”: iodine deficiency in industrialised countries, Proceedings of the Nutrition Society. (2010) 69, no. 1, 133–143, 10.1017/s0029665109991819. 19968908

[8] Sousa R. , Estações do ano, 2021, https://brasilescola.uol.com.br/geografia/estacoes-ano.htm.

[9] American Public Health Association , Standard Methods for the Examination of Water and Wastewater, 2005, 21, American Public Health Association, Washington, DC, USA.

[10] Dados climáticos para cidades mundiais, 2021, https://pt.climate-data.org/.

[11] Weather Spark , Clima e condições meteorológicas em Pinhais no ano todo, 2021, https://pt.weatherspark.com/y/29916/Clima-caracter%C3%ADstico-em-Pinhais-Brasil-durante-o-ano.

[12] Yang Z. , Wang C. , Nie Y. , Sun Y. , Tian M. , Ma Y. , Zhang Y. , Yuan Y. , and Zhang L. , Investigation on spatial variability and influencing factors of drinking water iodine in Xinjiang, China, PLoS One. (2021) 16, no. 12, e0261015, 10.1371/journal.pone.0261015. 34919574 PMC8682909

