# Correction to “Iodine Concentration in Drinking Water in the Same or Different Seasons of the Year in Brazilian Macroregions”

**DOI:** 10.1155/jnme/9825253

**Published:** 2026-07-19

C. A. Pinto, D. de Castro Morais, S. do Carmo Castro Franceschini, et al., “Iodine Concentration in Drinking Water in the Same or Different Seasons of the Year in Brazilian Macroregions,” *Journal of Nutrition and Metabolism*, 2022, 7227511, https://doi.org/10.1155/2022/7227511.

In the article, there were errors in the authorship. Mariana de Souza Macedo was omitted from the author list, and Fabiana de Cássia Carvalho Oliveira should not have been included in the author list.

Additionally, the name of Nathalia Marcolini Pelucio Pizato has been corrected.

The updated author contributions statement is below:

*“Carina Pinto and Silvia Priore designed the study; Carina Pinto carried out the study; Carina Pinto, Dayane Morais, and Silvia Priore analyzed and interpreted the data. Carina Pinto wrote the article. Mariana de Souza Macedo provided critical conceptual contributions, critically reviewed the manuscript, and approved the final version of the manuscript. All authors provided critical conceptual contributions and critically reviewed the manuscript. All authors approved the final manuscript for submission to the journal.”*

We apologize for these errors.

