# Supplementary material for: Iodine Concentration in Drinking Water in the Same or Different Seasons of the Year in Brazilian Macroregions
Source: J Nutr Metab. 2022 Aug 4;2022:7227511. doi: 10.1155/2022/7227511 (PMC11401693; doi:10.1155/2022/7227511)
Supplement: Supplementary file 1 — Supplementary Materials Two files are included in “Supplementary Files.” The files refer to the statistical analyses used to compare the concentration of iodine in drinking water between the climatic seasons of the same location and in the same climatic seasons between different locations. The file entitled “Results of Article Part 1‐Statistical Analysis” shows the comparison tests used to compare the concentration of iodine in drinking water between the climatic seasons of the same location. The other file entitled “Results of Article Part 2‐Statistical Analysis” shows the comparison tests used to compare the concentration of iodine in drinking water in the same climate stations between different locations in Brazilian macroregions. [file JNME-2022-7227511-s001.zip › 7227511.f1/ARTICLE RESULTS PART 2 - STATISTICAL ANALYSIS.doc]

**Article Results Part 2 - Statistical Analysis**

**Objective:** Compare the concentration of iodine in water between the same seasons of the year at different locations.

**Tabela 3:** Concentração mediana, mínima e máxima de iodo nas amostras de água coletadas nas Unidades Básicas de Saúde (UBS) durante a estação verão dos locais pertencentes às macrorregiões brasileiras.

| **Água de consumo** | **Locais/Regiões** | | | | | | | |
| --- | --- | --- | --- | --- | --- | --- | --- | --- |
|  | **Centro Oeste** | | **Nordeste** | | **Norte** | **Sudeste** | | **Sul** |
|  | **Brasília** | **Rondonópolis** | **Aracaju** | **São Luís** | **Palmas** | **Macaé** | **Viçosa** | **Pinhais** |
| Concentração mediana (µg⋅ L^-1^) | 4,43 | 2,33 | 3,55 | 0,00 | 0,41 | 3,85 | 3,33 | 13,04 |
| Concentração mínima (µg⋅ L^-1^) | 2,58 | 0,00 | 1,18 | 0,00 | 0,00 | 0,55 | 0,38 | 5,45 |
| Concentração máxima (µg⋅ L^-1^) | 18,58 | 10,59 | 5,91 | 4,88 | 2,68 | 24,18 | 28,24 | 42,59 |

**Brasília = 1, Rondonópolis = 2, Aracaju = 3, São Luís = 4, Palmas = 5, Macaé = 6, Viçosa = 7 e Pinhais = 8**

**Teste de Kurskal-Wallis - Resultado**

| **Estatísticas descritivas** | | | | | | | | |
| --- | --- | --- | --- | --- | --- | --- | --- | --- |
|  | N | Média | Desvio padrão | Mínimo | Máximo | Percentis | | |
|  |  |  |  |  |  | 25th | 50th (Median) | 75th |
| Conc_Iodo_Total | 90 | 5,5680 | 7,72953 | ,00 | 42,59 | ,8300 | 2,7200 | 6,5825 |
| Local_Cat | 90 | 4,53 | 2,304 | 1 | 8 | 2,75 | 4,00 | 7,00 |

| **Classificações** | | | |
| --- | --- | --- | --- |
|  | Local_Cat | N | Mean Rank |
| Conc_Iodo_Total | 1 | 10 | 58,80 |
|  | 2 | 12 | 32,00 |
|  | 3 | 12 | 48,67 |
|  | 4 | 12 | 20,29 |
|  | 5 | 10 | 18,60 |
|  | 6 | 9 | 54,61 |
|  | 7 | 14 | 53,79 |
|  | 8 | 11 | 78,64 |
|  | Total | 90 |  |

**Brasília = 1, Rondonópolis = 2, Aracaju = 3, São Luís = 4, Palmas = 5, Macaé = 6, Viçosa = 7 e Pinhais = 8**

| **Test Statistics^a,b^** | |
| --- | --- |
|  | Conc_Iodo_Total |
| Qui-quadrado | 48,252 |
| df | 7 |
| Significância Assintótica | ,000 |
| a. Kruskal Wallis Test | |
| b. Variável de agrupamento: Local_Cat | |

**Interpretação do Teste de Kruskal-Wallis: O teste de Kruskal-Wallis mostrou que há efeito do local sobre a concentração de iodo na água [X^2^ (7) = 48,252; p<0,05].**

**Fazendo as múltiplas comparações, dois a dois, chegamos aos seguintes resultados:**

**- Há diferença na concentração de iodo na água entre Rondonópolis (Região Centro Oeste) e Pinhais (Região Sul) (p=0,001).**

**- Há diferença na concentração de iodo na água entre São Luís (Região Nordeste) e Pinhais (Região Sul) (p=0,000).**

**- Há diferença na concentração de iodo na água entre São Luís (Região Nordeste) e Brasília (Região Centro Oeste) (p=0,015).**

**- Há diferença na concentração de iodo na água entre São Luís (Região Nordeste) e Viçosa (Região Sudeste) (p=0,030).**

**- Há diferença na concentração de iodo na água entre Palmas (Região Norte) e Pinhais (Região Sul) (p=0,000).**

**- Há diferença na concentração de iodo na água entre Palmas (Região Norte) e Brasília (Região Centro Oeste) (p=0,016).**

**- Há diferença na concentração de iodo na água entre Palmas (Região Norte) e Viçosa (Região Sudeste) (p=0,031).**

**Figura 1:** Colocar os gráficos de boxplot da concentração de iodo na água de consumo dos diferentes locais das macrorregiões brasileiras.

**Tabela 4:** Concentração mediana, mínima e máxima de iodo nas amostras de água coletadas nas Unidades Básicas de Saúde (UBS) durante a estação outono dos locais pertencentes às macrorregiões brasileiras.

| **Água de consumo** | **Locais/Regiões** | | | | | | |
| --- | --- | --- | --- | --- | --- | --- | --- |
|  | **Centro Oeste** | | **Nordeste** | | **Norte** | **Sudeste** | **Sul** |
|  | **Brasília** | **Rondonópolis** | **Aracaju** | **São Luís** | **Palmas** | **Viçosa** | **Pinhais** |
| Concentração mediana (µg⋅ L^-1^) | 3,81 | 0,00 | 2,76 | 0,00 | 0,00 | 2,49 | 5,45 |
| Concentração mínima (µg⋅ L^-1^) | 2,58 | 0,00 | 1,18 | 0,00 | 0,00 | 0,38 | 2,91 |
| Concentração máxima (µg⋅ L^-1^) | 6,28 | 113,86 | 5,91 | 1,93 | 2,68 | 12,20 | 11,35 |

**Brasília = 1, Rondonópolis = 2, Aracaju = 3, São Luís = 4, Palmas = 5, Viçosa = 6 e Pinhais = 7**

**Teste de Kruskal-Wallis – Resultado**

| **Estatísticas descritivas** | | | | | | | | |
| --- | --- | --- | --- | --- | --- | --- | --- | --- |
|  | N | Média | Desvio padrão | Mínimo | Máximo | Percentis | | |
|  |  |  |  |  |  | 25th | 50th (Median) | 75th |
| Conc_Iodo_Total | 81 | 4,2720 | 8,73634 | ,00 | 51,13 | ,0000 | 2,0700 | 4,6000 |
| Local_Cat | 81 | 4,06 | 1,983 | 1 | 7 | 2,00 | 4,00 | 6,00 |

| **Classificações** | | | |
| --- | --- | --- | --- |
|  | Local_Cat | N | Mean Rank |
| Conc_Iodo_Total | 1 | 10 | 54,90 |
|  | 2 | 12 | 33,17 |
|  | 3 | 12 | 50,25 |
|  | 4 | 12 | 16,17 |
|  | 5 | 10 | 19,70 |
|  | 6 | 14 | 45,29 |
|  | 7 | 11 | 67,82 |
|  | Total | 81 |  |

**Brasília = 1, Rondonópolis = 2, Aracaju = 3, São Luís = 4, Palmas = 5, Viçosa = 6 e Pinhais = 7**

| **Test Statistics^a,b^** | |
| --- | --- |
|  | Conc_Iodo_Total |
| Qui-quadrado | 44,334 |
| df | 6 |
| Significância Assintótica | ,000 |
| a. Kruskal Wallis Test | |
| b. Variável de agrupamento: Local_Cat | |

**Interpretação do Teste de Kruskal-Wallis: O teste de Kruskal-Wallis mostrou que há efeito do local sobre a concentração de iodo na água [X^2^ (6) = 44,334; p<0,05].**

**Fazendo as múltiplas comparações, dois a dois, chegamos aos seguintes resultados:**

**- Há diferença na concentração de iodo na água entre Rondonópolis (Região Centro Oeste) e Pinhais (Região Sul) (p=0,007).**

**- Há diferença na concentração de iodo na água entre Palmas (Região Norte) e Pinhais (Região Sul) (p=0,000).**

**- Há diferença na concentração de iodo na água entre Palmas (Região Norte) e Brasília (Região Centro Oeste) (p=0,014).**

**- Há diferença na concentração de iodo na água entre Palmas (Região Norte) e Aracaju (Região Nordeste) (p=0,044).**

**- Há diferença na concentração de iodo na água entre São Luís (Região Nordeste) e Pinhais (Região Sul) (p=0,000).**

**- Há diferença na concentração de iodo na água entre São Luís (Região Nordeste) e Brasília (Região Centro Oeste) (p=0,002).**

**- Há diferença na concentração de iodo na água entre São Luís (Região Nordeste) e Aracaju (Região Nordeste) (p=0,007).**

**- Há diferença na concentração de iodo na água entre São Luís (Região Nordeste) e Viçosa (Região Sudeste) (p=0,029).**

**Figura 2:** Colocar os gráficos de boxplot da concentração de iodo na água de consumo dos diferentes locais das macrorregiões brasileiras.

**Tabela 5:** Concentração mediana, mínima e máxima de iodo nas amostras de água coletadas nas Unidades Básicas de Saúde (UBS) durante a estação inverno dos locais pertencentes às macrorregiões brasileiras.

| **Água de consumo** | **Locais/Regiões** | | | | |
| --- | --- | --- | --- | --- | --- |
|  | **Centro Oeste** | | **Sudeste** | | **Sul** |
|  | **Brasília** | **Rondonópolis** | **Ribeirão Preto** | **Viçosa** | **Pinhais** |
| Concentração mediana (µg⋅ L^-1^) | 4,92 | 3,04 | 0,96 | 1,64 | 4,60 |
| Concentração mínima (µg⋅ L^-1^) | 2,39 | 1,30 | 0,09 | 0,38 | 2,91 |
| Concentração máxima (µg⋅ L^-1^) | 6,37 | 109,14 | 2,70 | 10,51 | 20,64 |

**Brasília = 1, Rondonópolis = 2, Ribeirão Preto = 3, Viçosa = 4 e Pinhais = 5**

**Teste de Kruskal-Wallis – Resultado**

| **Estatísticas descritivas** | | | | | | | | |
| --- | --- | --- | --- | --- | --- | --- | --- | --- |
|  | N | Média | Desvio padrão | Mínimo | Máximo | Percentis | | |
|  |  |  |  |  |  | 25th | 50th (Median) | 75th |
| Conc_Iodo_Total | 56 | 6,6823 | 15,36091 | ,09 | 109,14 | 1,3000 | 3,0400 | 5,6400 |
| Local_Cat | 56 | 3,07 | 1,412 | 1 | 5 | 2,00 | 3,00 | 4,00 |

| **Classificações** | | | |
| --- | --- | --- | --- |
|  | Local_Cat | N | Mean Rank |
| Conc_Iodo_Total | 1 | 10 | 35,20 |
|  | 2 | 12 | 35,42 |
|  | 3 | 9 | 10,78 |
|  | 4 | 14 | 21,86 |
|  | 5 | 11 | 37,82 |
|  | Total | 56 |  |

**Brasília = 1, Rondonópolis = 2, Ribeirão Preto = 3, Viçosa = 4 e Pinhais = 5**

| **Test Statistics^a,b^** | |
| --- | --- |
|  | Conc_Iodo_Total |
| Qui-quadrado | 20,446 |
| df | 4 |
| Significância Assintótica | ,000 |
| a. Kruskal Wallis Test | |
| b. Variável de agrupamento: Local_Cat | |

**Interpretação do Teste de Kruskal-Wallis: O teste de Kruskal-Wallis mostrou que há efeito do local sobre a concentração de iodo na água [X^2^ (4) = 20,446; p<0,05].**

**Fazendo as múltiplas comparações, dois a dois, chegamos aos seguintes resultados:**

**- Há diferença na concentração de iodo na água entre Ribeirão Preto (Região Sudeste) e Brasília (Região Centro Oeste) (p=0,011).**

**- Há diferença na concentração de iodo na água entre Ribeirão Preto (Região Sudeste) e Rondonópolis (Região Centro Oeste) (p=0,006).**

**- Há diferença na concentração de iodo na água entre Ribeirão Preto (Região Sudeste) e Pinhais (Região Sul) (p=0,002).**

**Figura 3:** Colocar os gráficos de boxplot da concentração de iodo na água de consumo dos diferentes locais das macrorregiões brasileiras.

**Tabela 6:** Concentração mediana, mínima e máxima de iodo nas amostras de água coletadas nas Unidades Básicas de Saúde (UBS) durante a estação primavera dos locais pertencentes às macrorregiões brasileiras.

| **Água de consumo** | **Locais/Regiões** | | | | |
| --- | --- | --- | --- | --- | --- |
|  | **Centro Oeste** | **Nordeste** | **Sudeste** | | **Sul** |
|  | **Rondonópolis** | **Aracaju** | **Ribeirão Preto** | **Viçosa** | **Pinhais** |
| Concentração mediana (µg⋅ L^-1^) | 3,04 | 3,55 | 0,09 | 3,75 | 7,98 |
| Concentração mínima (µg⋅ L^-1^) | 1,30 | 1,18 | 0,00 | 0,38 | 2,91 |
| Concentração máxima (µg⋅ L^-1^) | 13,48 | 8,28 | 4,45 | 17,26 | 20,64 |

**Rondonópolis = 1, Aracaju = 2, Ribeirão Preto = 3, Viçosa = 4 e Pinhais = 5**

**Teste de Kruskal-Wallis - Resultado**

| **Estatísticas descritivas** | | | | | | | | |
| --- | --- | --- | --- | --- | --- | --- | --- | --- |
|  | N | Média | Desvio padrão | Mínimo | Máximo | Percentis | | |
|  |  |  |  |  |  | 25th | 50th (Median) | 75th |
| Conc_Iodo_Total | 58 | 5,1909 | 4,57039 | ,00 | 20,64 | 1,8300 | 3,7300 | 7,3425 |
| Local_Cat | 58 | 3,00 | 1,439 | 1 | 5 | 2,00 | 3,00 | 4,00 |

| **Classificações** | | | |
| --- | --- | --- | --- |
|  | Local_Cat | N | Mean Rank |
| Conc_Iodo_Total | 1 | 12 | 30,92 |
|  | 2 | 12 | 30,25 |
|  | 3 | 9 | 9,56 |
|  | 4 | 14 | 28,50 |
|  | 5 | 11 | 44,73 |
|  | Total | 58 |  |

**Rondonópolis = 1, Aracaju = 2, Ribeirão Preto = 3, Viçosa = 4 e Pinhais = 5**

| **Test Statistics^a,b^** | |
| --- | --- |
|  | Conc_Iodo_Total |
| Qui-quadrado | 21,687 |
| df | 4 |
| Significância Assintótica | ,000 |
| a. Kruskal Wallis Test | |
| b. Variável de agrupamento: Local_Cat | |

**Interpretação do Teste de Kruskal-Wallis: O teste de Kruskal-Wallis mostrou que há efeito do local sobre a concentração de iodo na água [X^2^ (4) = 21,687; p<0,05].**

**Fazendo as múltiplas comparações, dois a dois, chegamos aos seguintes resultados:**

**- Há diferença na concentração de iodo na água entre Ribeirão Preto (Região Sudeste) e Rondonópolis (Região Centro Oeste) (p=0,041).**

**- Há diferença na concentração de iodo na água entre Ribeirão Preto (Região Sudeste) e Pinhais (Região Sul) (p=0,000).**

**Figura 4:** Colocar os gráficos de boxplot da concentração de iodo na água de consumo dos diferentes locais das macrorregiões brasileiras.
