# Supplementary material for: Iodine Concentration in Drinking Water in the Same or Different Seasons of the Year in Brazilian Macroregions
Source: J Nutr Metab. 2022 Aug 4;2022:7227511. doi: 10.1155/2022/7227511 (PMC11401693; doi:10.1155/2022/7227511)
Supplement: Supplementary file 1 — Supplementary Materials Two files are included in “Supplementary Files.” The files refer to the statistical analyses used to compare the concentration of iodine in drinking water between the climatic seasons of the same location and in the same climatic seasons between different locations. The file entitled “Results of Article Part 1‐Statistical Analysis” shows the comparison tests used to compare the concentration of iodine in drinking water between the climatic seasons of the same location. The other file entitled “Results of Article Part 2‐Statistical Analysis” shows the comparison tests used to compare the concentration of iodine in drinking water in the same climate stations between different locations in Brazilian macroregions. [file JNME-2022-7227511-s001.zip › 7227511.f1/ARTICLE RESULTS PART 1 - STATISTICAL ANALYSIS.doc]

**Article Results Part 1 - Statistical Analysis**

**Objective:** Compare the concentration of iodine in the water between the seasons of the year in the same place.

**Quadro 1** - Concentração mediana, mínima e máxima de iodo nas amostras de água de Brasília, referente as estações do ano.

| **Concentração de iodo (µg/L)** | **Estações do ano** | | | |
| --- | --- | --- | --- | --- |
|  | **Verão** | **Outono** | **Inverno** | **Primavera*** |
| Mediana | 4,43 | 3,81 | 4,92 | - |
| Mínima | 2,58 | 2,58 | 2,39 | - |
| Máxima | 18,58 | 6,28 | 6,37 | - |
| Diferença (Máx – Mín) | 16,00 | 3,70 | 3,98 | - |

*Não houve coleta de amostras de água na estação primavera.

**Teste de Kruskal-Wallis**

| **Estatísticas descritivas** | | | | | | | | |
| --- | --- | --- | --- | --- | --- | --- | --- | --- |
|  | N | Média | Desvio padrão | Mínimo | Máximo | Percentis | | |
|  |  |  |  |  |  | 25th | 50th (Median) | 75th |
| Conc_Iodo_Total | 30 | 5,1977 | 3,92919 | 2,39 | 18,58 | 2,5800 | 3,8100 | 5,8000 |
| Estacao_Ano | 30 | 2,00 | ,830 | 1 | 3 | 1,00 | 2,00 | 3,00 |

| **Classificações** | | | |
| --- | --- | --- | --- |
|  | Estacao_Ano | N | Mean Rank |
| Conc_Iodo_Total | 1 | 10 | 16,85 |
|  | 2 | 10 | 13,05 |
|  | 3 | 10 | 16,60 |
|  | Total | 30 |  |

| **Test Statistics^a,b^** | |
| --- | --- |
|  | Conc_Iodo_Total |
| Qui-quadrado | 1,202 |
| df | 2 |
| Significância Assintótica | ,548 |
| a. Kruskal Wallis Test | |
| b. Variável de agrupamento: Estacao_Ano | |

**Interpretação do Teste de Kruskal-Wallis: p>0,05 🡪 Não rejeito H0, logo não existe diferença na concentração de iodo na água entre as estações verão, outono e inverno (p=0,548).**

**Quadro 2** - Concentração mediana, mínima e máxima de iodo nas amostras de água de Macaé, referente as estações do ano.

| **Concentração de iodo (µg/L)** | **Estações do ano** | | | |
| --- | --- | --- | --- | --- |
|  | **Verão** | **Outono*** | **Inverno*** | **Primavera*** |
| Mediana | 3,85 | - | - | - |
| Mínima | 0,55 | - | - | - |
| Máxima | 24,18 | - | - | - |
| Diferença (Máx – Mín) | 23,63 | - | - | - |

*Não houve coleta de amostras de água nas estações outono, inverno e primavera.

**Quadro 3** - Concentração mediana, mínima e máxima de iodo nas amostras de água de Palmas, referente as estações do ano.

| **Concentração de iodo (µg/L)** | **Estações do ano** | | | |
| --- | --- | --- | --- | --- |
|  | **Verão** | **Outono** | **Inverno*** | **Primavera*** |
| Mediana | 0,41 | 0,00 | - | - |
| Mínima | 0,00 | 0,00 | - | - |
| Máxima | 2,68 | 2,68 | - | - |
| Diferença (Máx – Mín) | 2,68 | 2,68 | - | - |

*Não houve coleta de amostras de água nas estações inverno e primavera.

**Teste de Mann-Whitney**

| **Estatísticas descritivas** | | | | | | | | |
| --- | --- | --- | --- | --- | --- | --- | --- | --- |
|  | N | Média | Desvio padrão | Mínimo | Máximo | Percentis | | |
|  |  |  |  |  |  | 25th | 50th (Median) | 75th |
| Conc_Iodo_Total | 20 | ,6145 | 1,00418 | ,00 | 2,68 | ,0000 | ,0000 | ,8300 |
| Estacao_Ano | 20 | 1,50 | ,513 | 1 | 2 | 1,00 | 1,50 | 2,00 |

| **Classificações** | | | | |
| --- | --- | --- | --- | --- |
|  | Estacao_Ano | N | Mean Rank | Sum of Ranks |
| Conc_Iodo_Total | 1 | 10 | 11,60 | 116,00 |
|  | 2 | 10 | 9,40 | 94,00 |
|  | Total | 20 |  |  |

| **Test Statistics^a^** | |
| --- | --- |
|  | Conc_Iodo_Total |
| Mann-Whitney U | 39,000 |
| Wilcoxon W | 94,000 |
| Z | -,980 |
| Sig. Assint. (2 caudas) | ,327 |
| Sig exata [2*(Sig. de 1-cauda)] | ,436^b^ |
| a. Variável de agrupamento: Estacao_Ano | |
| b. Não corrigido para vínculos. | |

**Interpretação do Teste de Mann-Whitney: p>0,05 🡪 Não rejeito H0, logo não existe diferença na concentração de iodo na água entre as estações verão e outono (p=0,327).**

**Quadro 4** - Concentração mediana, mínima e máxima de iodo nas amostras de água de Pinhais, referente as estações do ano.

| **Concentração de iodo (µg/L)** | **Estações do ano** | | | |
| --- | --- | --- | --- | --- |
|  | **Verão** | **Outono** | **Inverno** | **Primavera** |
| Mediana | 13,04 | 5,45 | 4,60 | 7,98 |
| Mínima | 5,45 | 2,91 | 2,91 | 2,91 |
| Máxima | 42,59 | 11,35 | 20,64 | 20,64 |
| Diferença (Máx – Mín) | 37,14 | 8,44 | 17,73 | 17,73 |

**Teste de Kruskal-Wallis**

| **Estatísticas descritivas** | | | | | | | | |
| --- | --- | --- | --- | --- | --- | --- | --- | --- |
|  | N | Média | Desvio padrão | Mínimo | Máximo | Percentis | | |
|  |  |  |  |  |  | 25th | 50th (Median) | 75th |
| Conc_Iodo_Total | 44 | 9,8209 | 7,75343 | 2,91 | 42,59 | 4,7325 | 7,1300 | 11,3500 |
| Estacao_Ano | 44 | 2,50 | 1,131 | 1 | 4 | 1,25 | 2,50 | 3,75 |

**Verão = 1, Outono = 2, Inverno = 3 e Primavera = 4**

| **Classificações** | | | |
| --- | --- | --- | --- |
|  | Estacao_Ano | N | Mean Rank |
| Conc_Iodo_Total | 1 | 11 | 33,05 |
|  | 2 | 11 | 18,32 |
|  | 3 | 11 | 14,14 |
|  | 4 | 11 | 24,50 |
|  | Total | 44 |  |

| **Test Statistics^a,b^** | |
| --- | --- |
|  | Conc_Iodo_Total |
| Qui-quadrado | 13,631 |
| df | 3 |
| Significância Assintótica | ,003 |
| a. Kruskal Wallis Test | |
| b. Variável de agrupamento: Estacao_Ano | |

**Interpretação do Teste de Kruskal-Wallis: p<0,05 🡪 Rejeito H0, logo existe diferença na concentração de iodo na água entre as estações (verão, outono, inverno e primavera) (p=0,003).**

Fazendo as comparações múltiplas, dois a dois, chegamos aos seguintes resultados:

- Há diferença na concentração de iodo na água entre as estações outono e verão (p=0,041).

- Há diferença na concentração de iodo na água entre as estações inverno e verão (p=0,003).

**Quadro 5** - Concentração mediana, mínima e máxima de iodo nas amostras de água de Ribeirão Preto, referente as estações do ano.

| **Concentração de iodo (µg/L)** | **Estações do ano** | | | |
| --- | --- | --- | --- | --- |
|  | **Verão** | **Outono** | **Inverno** | **Primavera** |
| Mediana | - | - | 0,96 | 0,09 |
| Mínima | - | - | 0,09 | 0,00 |
| Máxima | - | - | 2,70 | 4,45 |
| Diferença (Máx – Mín) | - | - | 2,61 | 4,45 |

*Não houve coleta de amostras de água nas estações verão e outono.

**Teste de Mann-Whitney**

| **Estatísticas descritivas** | | | | | | | | |
| --- | --- | --- | --- | --- | --- | --- | --- | --- |
|  | N | Média | Desvio padrão | Mínimo | Máximo | Percentis | | |
|  |  |  |  |  |  | 25th | 50th (Median) | 75th |
| Conc_Iodo_Total | 18 | 1,1339 | 1,27031 | ,00 | 4,45 | ,0675 | ,9600 | 1,8300 |
| Estacao_Ano | 18 | 1,50 | ,514 | 1 | 2 | 1,00 | 1,50 | 2,00 |

| **Classificações** | | | | |
| --- | --- | --- | --- | --- |
|  | Estacao_Ano | N | Mean Rank | Sum of Ranks |
| Conc_Iodo_Total | 1 | 9 | 11,06 | 99,50 |
|  | 2 | 9 | 7,94 | 71,50 |
|  | Total | 18 |  |  |

| **Test Statistics^a^** | |
| --- | --- |
|  | Conc_Iodo_Total |
| Mann-Whitney U | 26,500 |
| Wilcoxon W | 71,500 |
| Z | -1,259 |
| Sig. Assint. (2 caudas) | ,208 |
| Sig exata [2*(Sig. de 1-cauda)] | ,222^b^ |
| a. Variável de agrupamento: Estacao_Ano | |
| b. Não corrigido para vínculos. | |

**Interpretação do Teste de Mann-Whitney: p>0,05 🡪 Não rejeito H0, logo não existe diferença na concentração de iodo na água entre as estações inverno e primavera (p=0,208).**

**Quadro 6** - Concentração mediana, mínima e máxima de iodo nas amostras de água de Rondonópolis, referente as estações do ano.

| **Concentração de iodo (µg/L)** | **Estações do ano** | | | |
| --- | --- | --- | --- | --- |
|  | **Verão** | **Outono** | **Inverno** | **Primavera** |
| Mediana | 2,33 | 0,00 | 3,04 | 3,04 |
| Mínima | 0,00 | 0,00 | 1,30 | 1,30 |
| Máxima | 10,59 | 113,86 | 109,14 | 13,48 |
| Diferença (Máx – Mín) | 10,59 | 113,86 | 107,84 | 12,18 |

**Teste de Kruskal-Wallis**

| **Estatísticas descritivas** | | | | | | | | |
| --- | --- | --- | --- | --- | --- | --- | --- | --- |
|  | N | Média | Desvio padrão | Mínimo | Máximo | Percentis | | |
|  |  |  |  |  |  | 25th | 50th (Median) | 75th |
| Conc_Iodo_Total | 48 | 9,1375 | 19,20462 | ,00 | 109,14 | ,8300 | 2,4700 | 8,2600 |
| Estacao_Ano | 48 | 2,50 | 1,130 | 1 | 4 | 1,25 | 2,50 | 3,75 |

**Verão = 1, Outono = 2, Inverno = 3 e Primavera = 4**

| **Classificações** | | | |
| --- | --- | --- | --- |
|  | Estacao_Ano | N | Mean Rank |
| Conc_Iodo_Total | 1 | 12 | 18,63 |
|  | 2 | 12 | 17,96 |
|  | 3 | 12 | 31,54 |
|  | 4 | 12 | 29,88 |
|  | Total | 48 |  |

| **Test Statistics^a,b^** | |
| --- | --- |
|  | Conc_Iodo_Total |
| Qui-quadrado | 9,651 |
| df | 3 |
| Significância Assintótica | ,022 |
| a. Kruskal Wallis Test | |
| b. Variável de agrupamento: Estacao_Ano | |

**Interpretação do Teste de Kruskal-Wallis: p<0,05 🡪 Rejeito H0, logo existe diferença na concentração de iodo na água entre as estações (verão, outono, inverno e primavera) (p=0,022).**

Fazendo as comparações múltiplas, dois a dois, chegamos aos seguintes resultados: não há diferença entre as estações ao compará-las duas a duas.

**Quadro 7** - Concentração mediana, mínima e máxima de iodo nas amostras de água de São Luís, referente as estações do ano.

| **Concentração de iodo (µg/L)** | **Estações do ano** | | | |
| --- | --- | --- | --- | --- |
|  | **Verão** | **Outono** | **Inverno*** | **Primavera** |
| Mediana | 0,00 | 0,00 | - | 0,19 |
| Mínima | 0,00 | 0,00 | - | 0,00 |
| Máxima | 4,88 | 1,93 | - | 2,07 |
| Diferença (Máx – Mín) | 4,88 | 1,93 | - | 2,07 |

*Não houve coleta de amostras de água na estação inverno.

**Teste de Mann-Whitney**

| **Estatísticas descritivas** | | | | | | | | |
| --- | --- | --- | --- | --- | --- | --- | --- | --- |
|  | N | Média | Desvio padrão | Mínimo | Máximo | Percentis | | |
|  |  |  |  |  |  | 25th | 50th (Median) | 75th |
| Conc_Iodo_Total | 24 | ,5583 | 1,22956 | ,00 | 4,88 | ,0000 | ,0000 | ,2925 |
| Estacao_Ano | 24 | 1,50 | ,511 | 1 | 2 | 1,00 | 1,50 | 2,00 |

| **Classificações** | | | | |
| --- | --- | --- | --- | --- |
|  | Estacao_Ano | N | Mean Rank | Sum of Ranks |
| Conc_Iodo_Total | 1 | 12 | 13,75 | 165,00 |
|  | 2 | 12 | 11,25 | 135,00 |
|  | Total | 24 |  |  |

| **Test Statistics^a^** | |
| --- | --- |
|  | Conc_Iodo_Total |
| Mann-Whitney U | 57,000 |
| Wilcoxon W | 135,000 |
| Z | -1,138 |
| Sig. Assint. (2 caudas) | ,255 |
| Sig exata [2*(Sig. de 1-cauda)] | ,410^b^ |
| a. Variável de agrupamento: Estacao_Ano | |
| b. Não corrigido para vínculos. | |

**Interpretação do Teste de Mann-Whitney: p>0,05 🡪 Não rejeito H0, logo não existe diferença na concentração de iodo na água entre as estações verão e outono (p=0,255).**

**Quadro 8** - Concentração mediana, mínima e máxima de iodo nas amostras de água de Viçosa, referente as estações do ano.

| **Concentração de iodo (µg/L)** | **Estações do ano** | | | |
| --- | --- | --- | --- | --- |
|  | **Verão** | **Outono** | **Inverno** | **Primavera** |
| Mediana | 3,33 | 2,49 | 1,64 | 3,75 |
| Mínima | 0,38 | 0,38 | 0,38 | 0,38 |
| Máxima | 28,24 | 12,20 | 10,51 | 17,26 |
| Diferença (Máx – Mín) | 27,86 | 11,82 | 10,13 | 16,88 |

**Teste de Kruskal-Wallis**

| **Estatísticas descritivas** | | | | | | | | |
| --- | --- | --- | --- | --- | --- | --- | --- | --- |
|  | N | Média | Desvio padrão | Mínimo | Máximo | Percentis | | |
|  |  |  |  |  |  | 25th | 50th (Median) | 75th |
| Conc_Iodo_Total | 56 | 4,3068 | 5,01072 | ,38 | 28,24 | 1,2200 | 2,9100 | 4,6000 |
| Estacao_Ano | 56 | 2,50 | 1,128 | 1 | 4 | 1,25 | 2,50 | 3,75 |

| **Classificações** | | | |
| --- | --- | --- | --- |
|  | Estacao_Ano | N | Mean Rank |
| Conc_Iodo_Total | 1 | 14 | 33,57 |
|  | 2 | 14 | 25,00 |
|  | 3 | 14 | 24,29 |
|  | 4 | 14 | 31,14 |
|  | Total | 56 |  |

| **Test Statistics^a,b^** | |
| --- | --- |
|  | Conc_Iodo_Total |
| Qui-quadrado | 3,357 |
| df | 3 |
| Significância Assintótica | ,340 |
| a. Kruskal Wallis Test | |
| b. Variável de agrupamento: Estacao_Ano | |

**Interpretação do Teste de Kruskal-Wallis: p>0,05 🡪 Não rejeito H0, logo não existe diferença na concentração de iodo na água entre as estações (verão, outono, inverno e primavera) (p=0,340).**

**Quadro 9** - Concentração mediana, mínima e máxima de iodo nas amostras de água de Aracaju, referente as estações do ano.

| **Concentração de iodo (µg/L)** | **Estações do ano** | | | |
| --- | --- | --- | --- | --- |
|  | **Verão** | **Outono** | **Inverno*** | **Primavera** |
| Mediana | 3,55 | 2,76 | - | 3,55 |
| Mínima | 1,18 | 1,18 | - | 1,18 |
| Máxima | 5,91 | 5,91 | - | 8,28 |
| Diferença (Máx – Mín) | 4,73 | 4,73 | - | 7,10 |

*Não houve coleta de amostras na estação inverno.

**Teste de Kruskal-Wallis**

| **Estatísticas descritivas** | | | | | | | | |
| --- | --- | --- | --- | --- | --- | --- | --- | --- |
|  | N | Média | Desvio padrão | Mínimo | Máximo | Percentis | | |
|  |  |  |  |  |  | 25th | 50th (Median) | 75th |
| Conc_Iodo_Total | 36 | 3,6572 | 1,73892 | 1,18 | 8,28 | 2,1675 | 3,5500 | 4,9250 |
| Estacao_Ano | 36 | 2,00 | ,828 | 1 | 3 | 1,00 | 2,00 | 3,00 |

| **Classificações** | | | |
| --- | --- | --- | --- |
|  | Estacao_Ano | N | Mean Rank |
| Conc_Iodo_Total | 1 | 12 | 16,83 |
|  | 2 | 12 | 15,79 |
|  | 3 | 12 | 22,88 |
|  | Total | 36 |  |

| **Test Statistics^a,b^** | |
| --- | --- |
|  | Conc_Iodo_Total |
| Qui-quadrado | 3,254 |
| df | 2 |
| Significância Assintótica | ,197 |
| a. Kruskal Wallis Test | |
| b. Variável de agrupamento: Estacao_Ano | |

**Interpretação do Teste de Kruskal-Wallis: p>0,05 🡪 Não rejeito H0, logo não existe diferença na concentração de iodo na água entre as estações (verão, outono e primavera) (p=0,197).**
